# Reproductive Ageing: Metabolic contribution to age-related chromosome missegregation in mammalian oocytes

**DOI:** 10.1530/REP-23-0510

**Published:** 2024-06-28

**Authors:** Bettina P Mihalas, Adele L Marston, Lindsay E Wu, Robert B Gilchrist

**Affiliations:** 1 Oocyte Biology Research Unit, Discipline of Women’s Health, School of Clinical Medicine, Faculty of Medicine and Health, UNSW Sydney, Kensington, Australia; 2 Wellcome Centre for Cell Biology, Institute of Cell Biology, School of Biological Sciences, University of Edinburgh, Edinburgh, United Kingdom; 3 School of Biomedical Sciences, Faculty of Medicine and Health, UNSW Sydney, Kensington, Australia

## Abstract

**In brief:**

Chromosome missegregation and declining energy metabolism are considered to be unrelated features of oocyte ageing that contribute to poor reproductive outcomes. Given the bioenergetic cost of chromosome segregation, we propose here that altered energy metabolism during ageing may be an underlying cause of age-related chromosome missegregation and aneuploidy.

**Abstract:**

Advanced reproductive age in women is a major cause of infertility, miscarriage and congenital abnormalities. This is principally caused by a decrease in oocyte quality and developmental competence with age. Oocyte ageing is characterised by an increase in chromosome missegregation and aneuploidy. However, the underlying mechanisms of age-related aneuploidy have not been fully elucidated and are still under active investigation. In addition to chromosome missegregation, oocyte ageing is also accompanied by metabolic dysfunction. In this review, we integrate old and new perspectives on oocyte ageing, chromosome segregation and metabolism in mammalian oocytes and make direct links between these processes. We consider age-related alterations to chromosome segregation machinery, including the loss of cohesion, microtubule stability and the integrity of the spindle assembly checkpoint. We focus on how metabolic dysfunction in the ageing oocyte disrupts chromosome segregation machinery to contribute to and exacerbate age-related aneuploidy. More specifically, we discuss how mitochondrial function, ATP production and the generation of free radicals are altered during ageing. We also explore recent developments in oocyte metabolic ageing, including altered redox reactions (NAD^+^ metabolism) and the interactions between oocytes and their somatic nurse cells. Throughout the review, we integrate the mechanisms by which changes in oocyte metabolism influence age-related chromosome missegregation.

## Introduction

Age-related female sub-fertility causes significant emotional stress on couples struggling to conceive and can impact the health of the next generation. This age-related decrease in fertility begins in a woman’s 30s and is caused by a decrease in oocyte quantity and quality (Thomas *et al.* 2021). The major contributor to the age-dependent decline in oocyte quality is chromosome missegregation or aneuploidy, which abruptly rises during a woman’s mid- to late 30s (Kuliev *et al.* 2011, Gruhn *et al.* 2019). These poor-quality oocytes increase the risk of chromosome disorders, including Down syndrome, Patau syndrome, sex chromosome disorders and Edwards syndrome (Kim *et al.* 2013, Cuckle & Morris 2021), and likely contribute to the increased time to conception, incidence of miscarriage and stillbirth in woman of advanced reproductive age (Nybo Andersen *et al.* 2000, Bateman & Simpson 2006). The age-related decline in oocyte quality is important as women in developed countries are delaying childbearing (Benzies 2008, Carolan & Frankowska 2011). In Australia, for example, the average childbearing age increased from 28.5 years in 1991 to 31.7 years in 2021, resulting in 25.5% of women having children over 35 (Australian Bureau of Statistics). This delay in childbearing is now a global phenomenon. Due to the decline in oocyte quality, advanced reproductive age is the largest defined cause of human infertility and a major reason for couples to be referred for assisted reproductive technologies (ARTs) (Fitzgerald & Catalgol 2017). Indeed, 64.4% of women seeking ART are over 35 (Newman 2022). Despite this, current ARTs are unable to rescue the age-related decline in oocyte quality or fertility, with live birth rates from IVF decreasing from 26.9% for women <35 to 16.6% for women between 35 and 39, and only 4.8% for women ≥40 (Newman 2022).

The biological origins of chromosome segregation defects in oocytes are beginning to be understood. Investigations have identified numerous alterations in chromosome segregation machinery, in older oocytes, including progressive loss of cohesin, microtubule instability and defects in the spindle assembly checkpoint (SAC) (Patel *et al.* 2015, Zielinska *et al.* 2015, Nakagawa & Fitzharris 2017, Blengini *et al.* 2021, Mihalas *et al.* 2024). Processes involved in chromosome segregation are intensely energy demanding. An important question is whether the ageing oocyte and its somatic cells are capable of adequately meeting the intense energy demands of oocyte meiosis, as there are also age-related changes in energy metabolism in the oocyte.

The topics of oocyte metabolism and age-related chromosome missegregation have been comprehensively discussed elsewhere (Richani *et al.* 2021, Wasielak-Politowska & Kordowitzki 2022). Here, this review will focus on the link between altered oocyte metabolism during ageing and chromosome missegregation, discussing potential metabolic mechanisms underlying the age-related increase in aneuploidy. This review will focus on mammalian oocytes, drawing mechanistic insights from other model organisms and from mitosis.

## Age-related chromosome missegregation

Meiosis is a complex process that requires carefully synchronised steps to ensure proper chromosome segregation, with unique aspects of this process in oocytes that make these cells particularly susceptible to age-related aneuploidy. Firstly, mammalian oocytes are formed in fetal ovaries as oogonia and then arrest at the diplotene stage of the first meiotic prophase as primordial follicles. These cells remain meiotically arrested in the ovary, even as they are recruited into the growing follicle pool until just prior to ovulation. Given that these non-renewable cells are arrested in a prolonged M-phase for decades, they can become highly susceptible to accumulated macromolecular damage that impacts their meiotic competence. Unlike mitosis, meiosis comprises only one cycle of DNA replication followed by two rounds of chromosome segregation to produce haploid gametes. Therefore, in mammals the loading of cohesin proteins, essential for the fidelity of chromosome segregation, occurs only once during S phase of fetal life, and these proteins must be retained until meiotic resumption potentially decades later (Tachibana-Konwalski *et al.* 2010, Burkhardt *et al.* 2016).

**Figure 1:**
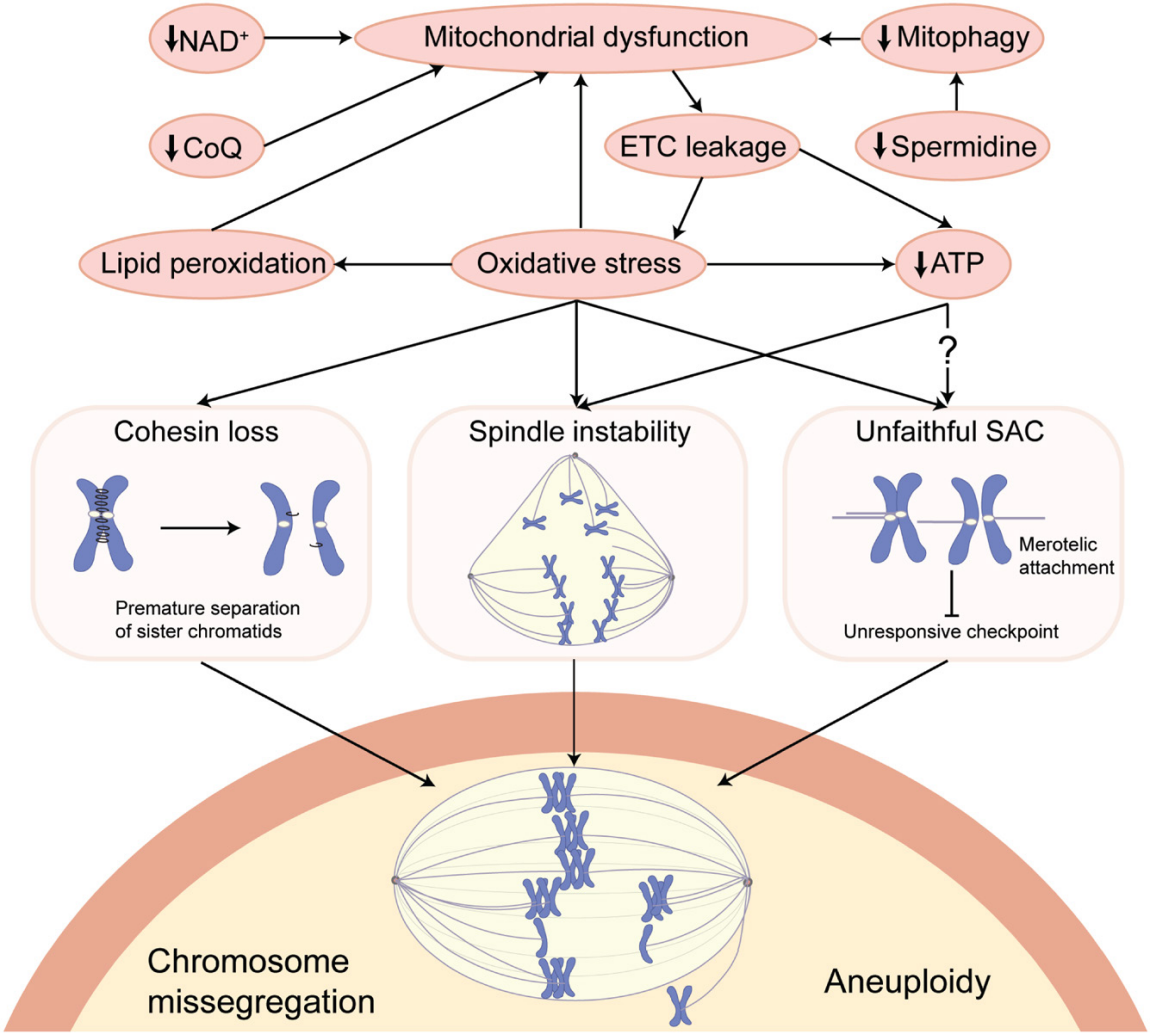
Metabolic mechanisms driving age-related chromosome missegregation in oocytes. Metabolic changes during ageing including declining NAD^+^, coenzyme Q10 (CoQ) and spermidine can impair mitochondrial function, resulting in electron transport chain (ETC) leakage and the formation of reactive oxygen species (ROS). This can lead to damage including lipid peroxidation and damage to long-lived proteins such as cohesin, which is required to prevent the premature separation of chromatids in the quiescent oocyte. Impaired mitochondrial function can also reduce ATP production, which is required for the bioenergetically demanding process of spindle assembly and chromosome segregation. Together, these metabolic changes with age are a potential cause of oocyte chromosome missegregation and aneuploidy. SAC, spindle assembly checkpoint.

Numerous alterations in chromosome segregation machinery have been identified in the ageing oocyte (Fig. 1). One of the most profound contributors to age-related oocyte aneuploidy is the progressive loss of the cohesin complex from chromosomes. Cohesin is responsible for maintaining cohesion between sister chromatids during the meiotic divisions. During ageing, there is a gradual decrease in meiotic cohesin on the chromosomes of mammalian eggs (Lister *et al.* 2010). Indeed, the cohesin that holds sister chromatids together throughout the prolonged prophase I arrest in primordial follicles is not replenished during the growing phase of oocytes, at least in mice (Tachibana-Konwalski *et al.* 2010). This means that the cohesin that is critical for proper chromosome segregation is the same cohesin that is laid down in the fetus, perhaps 40 years or earlier in humans. In addition, protection of cohesin by Shugoshin 2 during metaphase I is impaired in the oocytes of older women (Mihalas *et al.* 2024). This loss of cohesin leads to a decrease in cohesion between chromosomes, permitting the chromosomes to separate in a chaotic and untimely manner, increasing the incidence of aneuploidy (Cheng & Liu 2017). Importantly, in mitosis, cohesion between sister chromatids is resistant to loss of cohesin, with Carvalhal *et al*. (2018) only being able to induce premature separation of sister chromatids (PSSCs) after 80% of bound cohesin was removed (Carvalhal *et al.* 2018). It is tempting to speculate that progressive loss of cohesin in oocytes researches a critical point between the ages of 35 and 40 permitting PSSCs. Despite the critical role of the cohesin complex in maintaining chromosome cohesion and preventing aneuploidy, the underlying mechanisms of cohesin loss with age remain unknown. There are, however, strong hints in the literature to suggest that metabolic factors can influence the degree of cohesin loss, which we explore below.

Age-related aneuploidy as a result of cohesin loss may be further exacerbated by aberrant spindle forces. Faithful spindle formation in oocytes supports error-free meiosis (Holubcova *et al.* 2015, Nakagawa & Fitzharris 2017, Mogessie *et al.* 2018, Thomas *et al.* 2021). Chaotic spindle assembly and microtubule instability have been consistently observed in oocytes from woman of advanced reproductive age. Reciprocal transfer of nuclei between young and aged mouse oocytes have provided elegant evidence that the cytoplasm of the aged oocyte is responsible for generating altered microtubule dynamics, implicating altered spindle dynamics in old oocytes as an additional contributor to age-related chromosome missegregation during meiosis I (Nakagawa & Fitzharris 2017). To further support this, Dunkley and Mogessie 2023, demonstrated that age-related disruption of the cytoskeletal protein F-actin and subsequent disruption of microtubules exacerbated the premature splitting and scattering of sister chromatids when cohesin is reduced (Dunkley & Mogessie 2023). Given the finding that the aged cytoplasm can alter microtubule dynamics, the question is what factors present – or for that matter, absent – in the aged cytoplasm could impair spindle assembly. Here, we discuss a potential role for age-related alterations in oocyte metabolism as a cause for this decline.

Oocytes maintain delicate mechanisms that prevent premature progression through meiosis until their chromosomes are correctly attached to microtubules *via* kinetochores (Greaney *et al.* 2018). Briefly, the SAC sends a signal from kinetochores which inhibits entry into anaphase until microtubules are correctly attached (McAinsh & Kops 2023). Once correct kinetochore–microtubule attachments are established, the anaphase-promoting complex is activated, and separase carries out proteolysis to cleave the phosphorylated meiotic kleisin subunit of cohesin (Rec8), following which, chromosomes are separated (Duro & Marston 2015, Gryaznova *et al.* 2021, Nikalayevich *et al.* 2022). Uniquely, in meiosis, cohesin depletion must occur in two successive steps. During metaphase I (MI), the homologous chromosomes align on the spindle and at the onset of anaphase I, cohesin on chromosome arms is cleaved to resolve homologous chromosomes. This triggers the segregation of one set of homologous chromosomes into the first polar body. However, cohesin at the pericentromere is protected by the recruitment of the phosphate PP2A, so that sister chromatids remain held together. It is only following the alignment of sister chromatids on the spindle at metaphase II (MII) that the SAC is satisfied, and that cohesin at the pericentromere can be cleaved. This occurs upon fertilization, resulting in sister chromatid separation and segregation of one set into the second polar body generating a haploid gamete. There is mounting evidence indicating that fidelity of the SAC during meiosis I is compromised in oocytes during ageing (Marangos *et al.* 2015, Blengini *et al.* 2021), with insensitivity of the SAC persisting into meiosis II in aged mouse oocytes (Mihajlović *et al.* 2023). Mihajlović *et al.* (2023) recently demonstrated the SAC in aged oocytes failed to prevent the progression of meiosis II in the presence of misaligned chromosomes during meiosis II, which is consistent with observations of merotelic kinetochore attachment in oocyte meiosis II during oocyte ageing. In this scenario, a single kinetochore is pulled in opposing directions of a bipolar spindle, leading to segregation errors in sister chromatids (Cheng *et al.* 2017). It is important to consider that the insensitivity of the SAC in the old oocyte may not be consistent between mouse strains. Studies in CD1 and C57BL6 support the age-dependent insensitivity of the SAC, with no differences between meiotic timing or polar body (PB) extrusion rates between oocytes from young and old mice despite the prevalence of chromosome segregation errors (Yun *et al.* 2014, Suebthawinkul *et al.* 2023). In contrast, studies in the ICR mouse strain report decreased PB extrusion rates in oocytes from older mice (Miao *et al.* 2020, Zhang *et al.* 2023*b*). Potential stain variability highlights the importance of studying the SAC directly in human oocytes. Nevertheless, the cause of the permissive SAC in susceptible mouse strains is unclear.

One potential explanation for this age-related increase in chromosome missegregation could be the accompanying changes in energy metabolism in the oocyte that occur with age. The process of chromosome segregation, outlined above, is intensely energy demanding, with spikes in ATP production occurring at the resumption of meiosis I and at the point of first polar body extrusion (Dalton *et al.* 2014). Given this, age-related changes to oocyte metabolism could influence the integrity of the chromosome segregation machinery, which will be the subject of the rest of this review.

## Oocyte mitochondria and ATP generation

Oocytes are uniquely dependent on mitochondrial oxidative phosphorylation to generate ATP, as they are largely defective in glycolysis (Brinster 1971, Leese & Barton 1984), instead utilising pyruvate that is fed to the oocyte by the surrounding somatic cells (Leese & Barton 1985), which are capable of utilising glucose to complete glycolysis and to generate pyruvate on behalf of the oocyte (Downs, 1995). Dysregulation of mitochondrial processes have been universally reported to increase in cells with age, including oocytes (Chistiakov *et al.* 2014). A decrease in mitochondrial copy number (Chan *et al.* 2005, Simsek-Duran *et al.* 2013, Rambags *et al.* 2014), activity (Wilding *et al.* 2001), quality (Simsek-Duran *et al.* 2013, Rambags *et al.* 2014) and an increase in mutations to mitochondrial DNA (Barritt *et al.* 2000, Chan *et al.* 2005) have been well documented in mammalian oocytes with age, which have been comprehensively reviewed previously (Van Der Reest *et al.* 2021). Most recently, Smits *et al.* (2023) have conducted metabolic and lipidomic analysis in human cumulus cells and oocytes across different age groups, suggesting that this mitochondrial dysfunction also occurs in human reproductive ageing, with key alterations in NAD^+^, purine and pyrimidine pathways (Smits *et al.* 2023). There is also mounting evidence to suggest that the oocyte has a reduced capacity for mitophagy during ageing, with this turnover of aged or dysfunctional mitochondria being critical for meiotic competence (Cota *et al.* 2022, Jin *et al.* 2022, Khan *et al.* 2023), which may in part be overcome by restoring a decline in the levels of the polyamine metabolite spermidine (Zhang *et al.* 2023*b*).

In line with the increased rate of mitochondrial dysfunction in older oocytes, a corresponding decrease in ATP production has been reported in hamsters and mice (Simsek-Duran *et al.* 2013, Miao *et al.* 2020); however, there are conflicting findings on this. Smits *et al.* (2023) found no difference in ATP levels in human oocytes irrespective of age, instead reporting an age-related decrease in AMP and phosphocreatine in oocytes, which is associated with the adenosine salvage pathway, and an increase in ATP in the surrounding cumulus cells. These data suggest that alternative pathways for generating ATP may be invoked in the ageing oocyte (Smits *et al.* 2023).

Numerous studies have linked oocyte mitochondrial dysregulation to poor spindle assembly (Zhang *et al.* 2006, Ge *et al.* 2012, Zhang *et al.* 2023*a*). Recently, bioinformatics analysis from single-cell parallel methylation and transcriptome sequencing of young and old mouse oocytes revealed a strong correlation between gene expression signatures associated with mitochondrial dysfunction and abnormal spindle assembly (Zhang *et al.* 2023*a*). One hypothesis for the decrease in spindle integrity is that the energy supplied by dysfunctional mitochondria is insufficient to support spindle dynamics. During spindle assembly, polarised mitochondria dynamically relocalise, clustering around the forming spindle, presumably to support the increased demand for ATP (Van Blerkom & Runner 1984, Dalton *et al.* 2014, Takahashi *et al.* 2016, Al-Zubaidi *et al.* 2019). For example, the activity of kinesin motor proteins, which are essential for spindle assembly and migration to the oocyte’s cortex, is dependent on the hydrolysis of a significant amount of ATP (Camlin *et al.* 2017). Following from this, treatment with inhibitors of the electron transport chain that reduced ATP levels in oocytes led to the complete disassembly of the MI spindle (Zhang *et al.* 2006). Interestingly, loss of ATP, using the mitochondria inhibitor oligomycin A, was also able to reduce the sensitivity of the SAC in a mitotic cell line (Park *et al.* 2018). Upon ATP depletion, there was an increased association of the APC with the alternate activator protein CDH1, due to decreased translation of CDC20. This resulted in the degradation of cyclin B1, CDK1 inactivation and premature mitotic exit. It would also be interesting to explore whether this is similarly the case in meiosis, and which steps in this process that are rate limited by ATP levels, as kinase enzymes – including those involved in the regulatory steps of meiosis – are unlikely to become rate limited by ATP concentrations, which in most cases exceed the K_M_ of these enzymes by at least ten-fold (Bennett *et al.* 2009, Park *et al.* 2016).

Another aspect of spindle assembly that could be susceptible to altered energy homeostasis is the requirement for guanosine triphosphate (GTP) in spindle assembly during meiosis (Caplow & Shanks 1990, Cesario & Mckim 2011, Drutovic *et al.* 2020, Shemesh *et al.* 2023). The energy required for spindles to physically pull sister chromatids in opposing directions is derived from the lattice energy that is stored in tubulin microtubules (Caplow *et al.* 1994, Vulevic & Correia 1997), whose initial assembly is fuelled by the hydrolysis of GTP by Ras-related nuclear protein (Ran-GTP) (Caplow & Shanks 1990, Shemesh *et al.* 2023). Concentration gradients of Ran-GTP surrounding the chromosome (Oh *et al.* 2016) are essential to co-ordinate spindle formation and accurate chromosome segregation (Carazo-Salas *et al.* 2001, Kalab *et al.* 2002, Holubcova *et al.* 2015), and it is conceivable that declining GTP levels could impair this process. While a decrease in GTP has not been directly demonstrated in oocytes during ageing, its precursor, guanosine monophosphate, was shown to be significantly reduced in human MI oocytes of advanced reproductive age (Smits *et al.* 2023). Further, GTP production is mediated by succinyl CoA synthetase, a key enzyme of the TCA cycle which converts succinyl CoA into succinate and, in doing so, phosphorylates GDP into GTP. This crucial step of the TCA cycle takes place in the mitochondria, and it is conceivable that declining mitochondrial function with increased age could impair the GTP required for accurate spindle assembly and chromatid segregation.

One other aspect through which impaired GTP production could alter oocyte function is in the energy requirements for accurate protein translation. During early follicle development, oocytes drastically expand in size, with commensurate biosynthetic requirements for protein translation. Recently, it was found that transcriptional elongation speed accelerates with biological age, resulting in a greater rate of transcriptional errors (Debes *et al.* 2023). Interestingly, of the genes that have the greatest increase in transcriptional elongation speed are genes involved in metabolism and catabolism (Debes *et al.* 2023). In line with this, oocytes from aged animals have altered ribosomal machinery (Duncan *et al.* 2017).

Overall, despite some conflicting literature, it does seem logical to hypothesise that defective mitochondria in the ageing oocyte may not provide a sufficient, localised supply of energy to support proper spindle assembly and SAC function, ultimately leading to oocyte aneuploidies.

## Oxidative stress

While impaired ATP generation could impact spindle assembly during ageing, another consequence of mitochondrial dysfunction in oocytes could be related to the increased production of reactive oxygen species (ROS). Under experimental conditions, 0.2% of the O_2_ consumed in respiration is reduced to superoxide (O_2_^−^) as a by-product of generating ATP through the electron transport chain (Herrero & Barja 1997, St-Pierre *et al.* 2002, Kudin *et al.* 2004). Physiological production of O_2_^−^ is likely to be even lower; however, precise measurements of this is challenging. Despite this, the prolonged life of the non-renewable ovarian reserve could mean that over the decades between oogenesis in the developing fetus and ovulation in an adult, O_2_^−^ production at even low levels could lead to the accumulation of cellular damage. To overcome this, quiescent oocytes in ovarian primordial follicles are maintained with their mitochondria in an unusual state, where they are deficient in complex I of the electron transport chain (Rodriguez-Nuevo *et al.* 2022), which is the primary site of ROS generation in the mitochondria (Kudin *et al.* 2004). Respiratory complexes of the electron transport chain are large, multi-subunit complexes that require the delicate assembly of individual proteins that are encoded by both the nuclear and mitochondrial genomes, requiring tight coordination of transcriptional activity in both genomes. Failure to do so can lead to an incorrect stoichiometry in the production of these subunits, impacting mitochondrial proteostasis (Houtkooper *et al.* 2013) and triggering the mitochondrial unfolded protein response (Zhao *et al.* 2002). In addition, dysfunctional complex assembly can lead to electron transport chain leakage and the production of O_2_^−^. One essential component to electron transport chain function is co-enzyme Q10 (coQ10), otherwise known as ubiquinone, which mediates the transfer of electrons from complex I and II to complex III. The absence of this factor can lead to electron leakage and ROS formation, while exogenous supplementation with coQ10 can reduce ROS, restore ATP production and spindle integrity, and delay reproductive ageing, suggesting a decrease in oocyte coQ10 with age (Ben-Meir *et al.* 2015).

The decline in oocyte mitochondrial integrity during reproductive ageing (Zhang *et al.* 2023*a*) is associated with an increase in oxidative stress in mammalian oocytes (Smits *et al.* 2023) which has ongoing deleterious effects on oocyte quality, inflicting DNA, lipid and protein damage (Mihalas *et al.* 2017*b*). Furthermore, mitochondria are susceptible to further damage by ROS (Van Der Reest *et al.* 2018), potentially leading to a self-perpetuating cascade of oxidative stress (Van Der Reest *et al.* 2018). Indeed, exposure of mouse oocytes to an acute oxidative insult leads to a loss in mitochondrial membrane potential and a decrease in ATP levels (Zhang *et al.* 2006). Further, in mouse oocytes, the mitochondrial protein, succinate dehydrogenase (SDHA), is susceptible to modification by the lipid peroxidation byproduct, 4-hydroxynonenal (4HNE) and was proposed to stimulate leakage from the electron transport train (Lord *et al.* 2015). Importantly, increased generation of ROS is further exacerbated by the decreased capacity of ageing oocytes to resolve oxidative stress and repair oxidative damage (Mihalas *et al.* 2017*b*). Indeed, although ovarian ROS damage accumulates with age, the impairment in mitochondrial function might only be one cause, as ovarian ageing in non-human primates is also associated with reduced antioxidant gene expression (Wang *et al.* 2020).

Oxidative stress has been linked to chromosome missegregation in meiosis (Fig. 1). Indeed, in mouse models, oxidative stress, including H_2_O_2_ and 4HNE, has been shown to increase the incidence of aneuploidies caused by PSSC (Mihalas *et al.* 2017*a*). In *Drosophila* oocytes, elevated levels of oxidative stress caused by conditional knockdown of ROS scavenger enzymes (SOD1 and SOD2) have been shown to induce premature loss of cohesion and subsequent chromosome segregation errors (Perkins *et al.* 2016). Prolonged suppression of ovulation, which requires ROS production (Shkolnik *et al.* 2011), directly prevented the loss of cohesin in oocytes from aged mice (Chatzidaki *et al.* 2021), although ROS was not examined in this study. While treatment with exogenous ROS insults reduces sister chromatid cohesion (Perkins *et al.* 2016, Mihalas *et al.* 2017*a*), the mechanism by which this impacts the cohesin complex warrants further investigation.

As with chromosome segregation errors and loss of the cohesin complex, oxidative stress has also been linked to altered spindle integrity. In terms of mechanism, one hypothesis is that oxidative stress causes mitochondrial damage, reducing ATP production required for spindle assembly and stability, with exogenous ROS insults induce mitochondrial damage and decreased cellular ATP in young mouse oocytes (Zhang *et al.* 2006). With age, there is an accumulation of lipid peroxidation and 4HNE production (Mihalas *et al.* 2017*a*, Smits *et al.* 2023), which can covalently modify the alpha-, beta-, and gamma-tubulin proteins (Mihalas *et al.* 2017*a*), impairing tubulin polymerisation (Stewart *et al.* 2007) and inducing aneuploidy in oocytes. Lipid peroxidation and the accumulation of these by-products increase in mouse and human oocytes with age (Mihalas *et al.* 2017*a*, Smits *et al.* 2023), and 4HNE-modified amino acids identified on alpha and beta tubulin are consistent with altered polymerisation (Stewart *et al.* 2007).

Desensitisation of the SAC in oocytes in response to age-related oxidative stress is also likely. Recently, Blengini *et al.* (2021) identified a reduced expression of the kinetochore-associated SAC proteins MAD2, ZW10 and securin in mouse oocytes in response to increased ROS induced by the oocyte-specific deletion of AURKB (Blengini *et al.* 2021). The authors propose that this increase in ROS may perturb protein homeostasis to reduce the expression of SAC proteins. Simultaneously, accumulating evidence from studies of somatic cells suggests that even slightly increased concentrations of H_2_O_2_ may undermine the sensitivity of the SAC (D’angiolella *et al.* 2007, Goutas *et al.* 2023). Hence, oocyte oxidative stress could be a culprit contributing to chromosome missegregation and subsequent aneuploidies in oocytes and embryos from older females.

Lastly, ROS have also been implicated in age-related telomere length shortening which is observed in mammalian oocytes (Yamada-Fukunaga *et al.* 2013). In cancer cells, telomere shortening is associated with genomic instability and aneuploidy whereby free chromosome ends lead to telomere fusions and genomic rearrangements during cell division (Cleal *et al.* 2018). In oocytes, telomere shortening in telomerase null mice resulted in abnormal chromosome alignment and meiotic spindles at MI and MII in the fourth generation (Liu *et al.* 2002). There is also some evidence to suggest that telomere shortening is associated with aneuploidy in human oocytes and early pre-implantation embryos, with aneuploid polar bodies having significantly less telomere DNA than euploid sibling oocytes (Treff *et al.* 2011).

To add another layer of complexity, work in mitotic cells has also demonstrated that telomere shortening regulates mitochondrial function. Indeed, Sahin *et al.* first reported that telomere dysfunction leads to p53-mediated repression of the mitochondrial regulators peroxisome proliferator-activated receptor gamma, coactivator 1 alpha and beta, resulting in impaired mitochondrial function, biogenesis and increased ROS (Sahin *et al.* 2011). An alternative hypothesis for why telomere shortening impairs mitochondrial function is that the activation of DNA repair pathways leads to the consumption of nicotinamide adenine dinucleotide (NAD^+^) which is an essential co-factor for mitochondrial respiration (Fang *et al.* 2014) and is discussed in more detail in later sections.

Although an extensive body of literature has over many decades investigated the idea that excess ROS production in oocytes is a cause of female infertility, there are several lines of evidence that argue against this. Firstly, ROS can act as intracellular signalling intermediates, with a crucial role in the resumption of meiosis (Pandey *et al.* 2010, Tiwari & Chaube 2016) and fertilisation (Han *et al.* 2018). Ovulation is also dependent on ROS production and can be blocked through treatment with antioxidants (Shkolnik *et al.* 2011). Despite prolonged interest in this idea, the totality of clinical trial evidence for antioxidants is uncertain, with mixed findings from a base of very low-quality evidence (Showell *et al.* 2020). While the free radical theory of ageing was previously a popular concept in the biology of ageing (Harman 2006), since its inception in 1954, clinical trials for antioxidants have comprehensively failed to show any impact on human mortality (Bjelakovic *et al.* 2012). Not only have these compounds failed to demonstrate an impact in the clinic, they actively block both the metabolic benefits of exercise (Ristow *et al.* 2009) and the benefits of calorie restriction on lifespan (Schulz *et al.* 2007). The free radical theory has now been largely rejected, at least in the field of ageing research (Gladyshev 2014, Stuart *et al.* 2014). Given this, what other metabolic mechanisms could explain the decline in spindle assembly and accurate chromosome segregation with age?

## Metabolic co-factor availability: NAD^+^

Another potential mechanism that could impair the bioenergetic requirements for spindle assembly could be a decline in levels of redox cofactors such as NAD^+^/NADH and flavin adenine dinucleotide (FAD^+^/FADH_2_), which are essential for diverse biochemical reactions required for cell metabolism. NAD^+^ acts as a metabolic lubricant for the exchange of electrons between enzyme-mediated reactions in glycolysis, β-oxidation and the TCA cycle. Deficiency of NAD^+^ can severely alter metabolic flux (Tan *et al.* 2015), with a reduction in glycolysis and subsequent TCA cycle activity downstream of the enzyme glyceraldehyde-3-phosphate dehydrogenase (G3PDH), the first enzyme in this pathway that requires NAD^+^ as a cofactor. Levels of this metabolite decline with age, including in oocytes and the ovary in mice (Bertoldo *et al.* 2020, Miao *et al.* 2020, Yang *et al.* 2020) and in humans (Smits *et al.* 2023). This decline can be reversed using endogenous precursors to NAD^+^ biosynthesis, including nicotinamide mononucleotide (NMN) and nicotinamide riboside (NR), both of which, notably, restored age-related defects in spindle assembly and overall fertility (Bertoldo *et al.* 2020, Miao *et al.* 2020, Yang *et al.* 2020). Bertoldo *et al*. (2020) demonstrated that just 4 weeks of oral supplementation with NMN was sufficient to improve spindle assembly, embryo quality and ultimately fertility in old mice (Bertoldo *et al.* 2020). Similarly, daily intraperitoneal injection of NMN for just 10 days was able to improve aberrant spindles, misaligned chromosomes, incorrect kinetochore–microtubule attachments, aneuploidy and embryo development in mouse oocytes. The authors attributed this to the recovery of mitochondrial activity and the subsequent increased ATP and decrease in ROS (Miao *et al.* 2020). Supplementation of NAD^+^ precursors to the oocyte during *in vitro* maturation has also been met with some success in improving, oocyte maturation, spindle formation and embryo development from oocytes of young animals but has not yet been explored in the context of chromosome segregation and ageing (Pollard *et al.* 2021, Pollard *et al.* 2022, El Sheikh *et al.* 2020). These impacts from NAD^+^ precursor treatment could be related to the need for NAD^+^ biosynthesis in maintaining spindle dynamics, in particular the asymmetry required for extrusion of the PB (Wei *et al.* 2020). Acute silencing or small molecule inhibition of the NAD^+^ biosynthetic enzyme NAMPT slows the migration of spindles from the midzone to the oocyte cortex, with the resulting inability to properly extrude the PB which causes membrane ingress around the spindle, with invagination of ooplasmic material and eventual release of a larger PB (Wei *et al.* 2020). Most strikingly, this enzyme co-localised with the mitochondria around the spindle, while NAMPT inhibition abolished not just NAMPT localisation but the clustering of mitochondria around the spindle. This supports the idea that NAD^+^ production is essential to mitochondrial function and the bioenergetics of spindle assembly.

It is also possible that these age-related changes in NAD^+^ metabolism could also contribute to oocyte aneuploidy by affecting chromatin architecture and the expression of genes involved in chromosome segregation. In mammalian oocytes, the N-termini of H3 and H4 are globally deacetylated at MI and MII (Endo *et al.* 2005, Kim *et al.* 2003, Akiyama *et al.* 2004). Chemical inhibition of histone deacetylation during meiosis led to aberrant chromosomal arrangement and aneuploidy in one cell zygotes (Akiyama *et al.* 2006). As oocytes have low rates of transcription during meiotic resumption, it is possible that increased aneuploidy from inhibition of histone deacetylation at this stage is attributed to changes in chromatin architecture rather than gene expression. Notably, oocytes from older animals were also shown not to undergo meiotic histone deacetylation efficiently (Akiyama *et al.* 2006), and in human oocytes, residual acetylation at H4K12, which is normally deacetylated during meiotic maturation (Zhang *et al.* 2020), was more frequent with increasing maternal age and associated with chromosome misalignment (Van Den Berg *et al.* 2011). NAD^+^ is used as a cofactor by the sirtuin class of deacetylase enzymes (Imai *et al.* 2000) and the role of these proteins in meiosis have been comprehensively reviewed elsewhere (Vazquez *et al.* 2020). Transgenic overexpression of SIRT2 was shown to decrease spindle abnormalities, aneuploidy and oxidative stress in oocytes of older animals; however, spindle assembly in SIRT2 knockout animals was normal (Bertoldo *et al.* 2020). While it may be tempting to attribute the decline in histone deacetylation with age to the decline in levels of a cofactor for the sirtuin enzymes, future work should aim to measure the absolute concentration of NAD^+^ in oocytes, to compare these to the known K_M_ of sirtuins for NAD^+^, which will determine whether the decline in NAD^+^ with age is physiologically relevant to sirtuin activity. A key challenge, however, is bioanalytical sensitivity using mass spectrometry due to the low volume obtained from individual oocytes, with previous assays for oocyte NAD^+^ instead using relative measurements such as autofluorescence (Bertoldo *et al.* 2020, Campbell *et al.* 2022).

In line with the idea that NAD^+^ changes with age could alter histone acetylation and gene expression, single-cell transcriptomics showed that *in vivo* treatment of reproductively aged mice with NMN resulted in an altered transcriptional profile (Miao *et al.* 2020). This would be consistent with an altered epigenetic landscape; however, this was not measured in that study.

NAD^+^ is also a co-factor for poly-(ADP) ribose polymerase enzymes (PARPs), which are essential for regulating DNA repair, chromatin architecture and gene expression. Poly-ADP-ribosylation (PARylation) is enriched at the cortical areas of oocytes during meiosis, where it is essential to polar body extrusion (Xie *et al.* 2018). Further, inhibition of PARP activity using pharmacological agents can deplete the ovarian reserve (Winship *et al.* 2020). It is possible that declining NAD^+^ levels in the older oocyte could impair oocyte function by depleting substrate availability for the PARP enzymes.

Taken together, it is logical to hypothesise that age-related changes to NAD^+^ metabolism influence gene expression; however, the extent to which NAD^+^ metabolism regulates oocyte epigenetics remains largely speculative and warrants deeper investigation.

## Communication between the somatic cells and the oocyte

As discussed above, oocyte metabolism is critically linked to the function of their somatic nurse cells, which can replace metabolic processes that oocytes may be deficient in such as glycolysis (Richani *et al.* 2021). Granulosa cells surround oocytes throughout ovarian follicle development, proliferating to form multiple layers that encapsulate the growing oocyte, forming the granulosa–oocyte complex. As the follicle and oocyte increase in size, paracrine factors secreted by the oocyte trigger the differentiation of the granulosa cells that directly surround the oocyte into cumulus cells (Li *et al.* 2000), forming the cumulus–oocyte complex (COC). This complex is maintained at all stages throughout antral follicle development and meiotic maturation until soon after fertilisation. Surrounding somatic cells physically connect to the oocyte via transzonal projections (TZPs), which facilitate the bi-directional trafficking of regulatory factors and metabolites that are essential to oocyte function. The reliance of an oocyte on its surrounding somatic cells for the acquisition of developmental competence has been consistently demonstrated. Indeed, oocyte-specific transgenic inhibition of clathrin-mediated endocytosis during follicle development led to the complete loss of antral follicles due to oocyte apoptosis (Mihalas *et al.* 2020). The removal of cumulus cells, or inhibition of gap junctions, results in perturbed oocyte metabolism, fertilisation and embryo development (Richani *et al.* 2021). This is likely, at least partly, due to the provision of pyruvate and lactate as substrates for ATP generation by oxidative phosphorylation as described earlier, as oocytes are unable to carry out glycolysis and rely on a supply of these metabolites from supporting cells (Downs, 1995).

**Figure 2:**
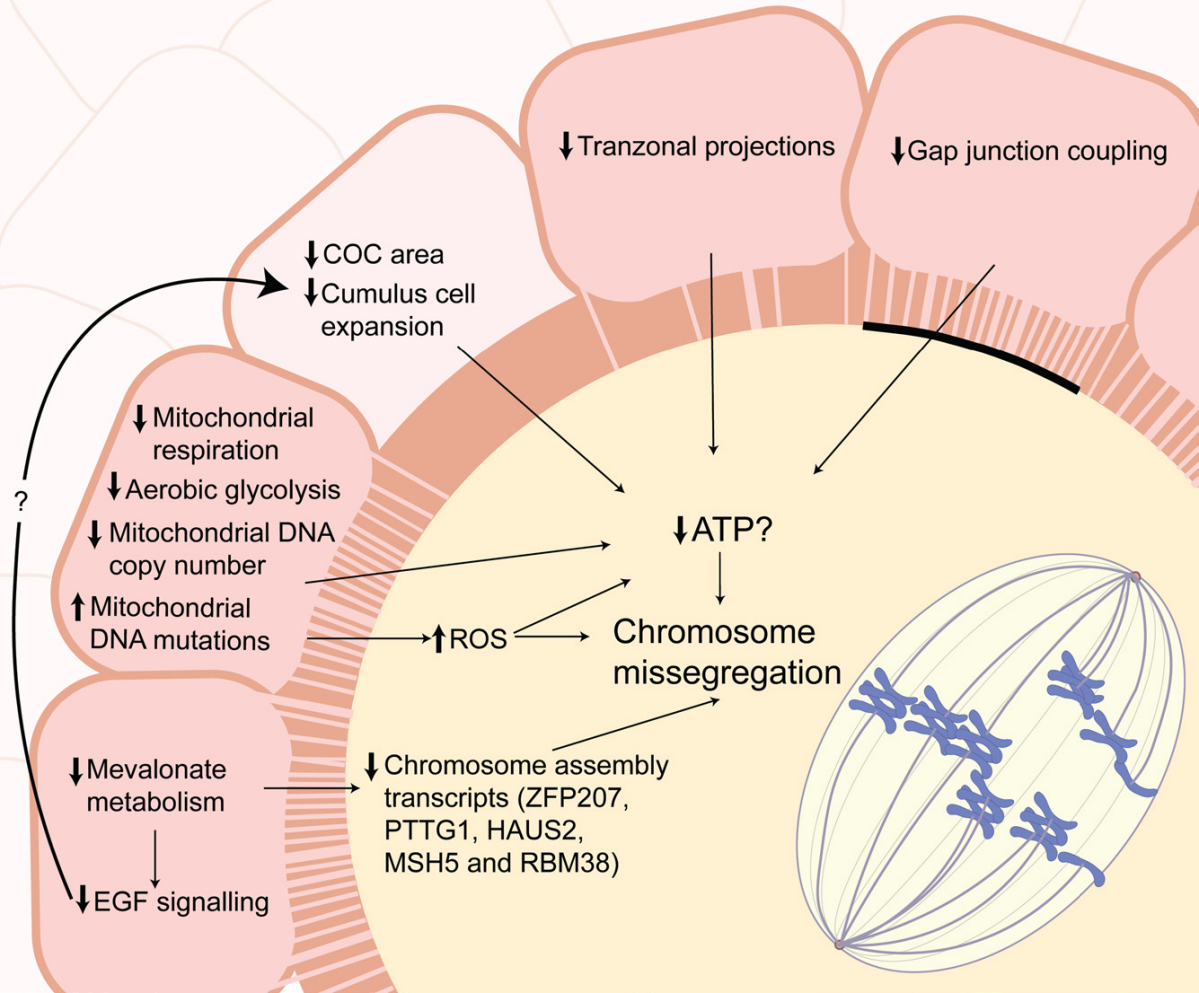
Compromised somatic cell support to the oocyte as a cause of chromosome missegregation with age. Granulosa cells and cumulus cells act as somatic nurse cells, extending to the oocyte through transzonal projections (TZPs) that culminate in gap junctions in the oocyte membrane for the transport of critical metabolites such as pyruvate, due to the inability of the oocyte to perform glycolysis. TZP communication to the oocyte is impaired with age, potentially limiting ATP production needed to fuel bioenergetically demanding processes related to spindle assembly and chromosome segregation. During ageing, there is a decrease in cumulus cell expansion. Somatic support cells are also susceptible to age-related metabolic defects in glycolysis, mitochondrial function, and mevalonate metabolism, which can impact epidermal growth factor (EGF) signalling and the transcription of proteins essential to chromosome assembly.

Blocking the transport of these factors from the somatic ovarian cells to the oocyte likely impairs transport of various metabolites in oocytes as has been demonstrated for the case of ATP (Dalton *et al.* 2014, Richani *et al.* 2019). There is evidence that communication between somatic ovarian cells and oocytes is compromised during ageing (Fig. 2). In one study in mice, the number of TZPs in COCs from old mice was reduced by approximately 40%, resulting in a decrease in gap junctional coupling (El-Hayek *et al.* 2018). One hypothesis is that a decrease in intra-COC communication may be a consequence of the structural remodelling that occurs in the ovary during ageing (Briley *et al.* 2016, Amargant *et al.* 2020, Umehara *et al.* 2022). One question for the field is whether declining oocyte quality is a cell-autonomous process, or whether it is in fact a decline in the function of the supporting somatic cells that drives reproductive ageing. Recently, Babayev *et al.* (2023) identified a reduction in COC area in immature oocytes and further noted a decrease in cumulus cell expansion in mice of advanced reproductive age, which was attributed to a loss in matrix integrity and hyaluronan production (Babayev *et al.* 2023). When considering the importance of oocyte signalling in orchestrating cumulus cell differentiation and expansion, it remains unclear whether decreased cumulus cell density and expansion with age is a cause or a consequence of decreased COC communication. The functional impact of decreased somatic cell communication with the oocyte is highlighted by the recently proposed hypothesis that the older oocyte may have an increased dependence on the surrounding cumulus cells. Indeed, Smits *et al.* 2023 suggest that increased ATP in the surrounding cumulus cells may compensate for mitochondrial dysfunction in the ageing oocyte (Smits *et al.* 2023).

The importance of oocyte–somatic cell metabolite exchange extends beyond just bioenergetics. The mevalonate pathway is important for the production of cholesterol and sterol biosynthesis, which in the ovary is critical to the production of steroid hormones. This pathway is dysregulated in granulosa cells in mouse and human reproductive ageing (Blengini & Schindler 2023, Liu *et al.* 2023) and contributes to the increased rate of aneuploidy. Inhibition of mevalonate metabolism in COCs during* in vitro* maturation (IVM) resulted in aneuploidy in the oocytes of young mice. In line with this, supplementation of geranylgeraniol, an intermediate in this pathway, to COCs during IVM or through intraperitoneal injection, reduced meiotic defects in old oocytes, recovering maturation rates, spindle integrity and decreasing aneuploidy (Liu *et al.* 2023). The authors further demonstrate that mevalonate metabolism is important for EGF signalling and the expression of meiosis-associated transcripts related to chromosome assembly. Curiously, there was no effect of manipulation of mevalonate metabolism, through inhibition or supplementation, on the meiotic integrity of denuded oocytes, supporting the notion that dysregulated metabolism of cumulus cells may influence downstream chromosome segregation machinery of oocytes.

An alternative and provocative interpretation of the role of somatic cell interactions has suggested that the retraction of TZPs with age is orchestrated by the oocyte to mitigate damage from the ageing somatic environment (Alberico & Woods 2021). Numerous studies have reported alternations in granulosa and cumulus cell quality with age (Babayev & Duncan 2022). Most notably, aged somatic supporting cells are susceptible to distinct metabolic alterations. For instance, luteinised granulosa cells of women of advanced reproductive age show lower mitochondrial respiration, aerobic glycolysis and a decrease in their cellular energy charge (ATP/ADP or AMP ratio) (Cecchino *et al.* 2021). Further, cumulus cells are associated with lower mitochondrial DNA copy number and increased mutations (Tsai *et al.* 2010, Yang *et al.* 2021). A study by Perez and Tilly also support this hypothesis, demonstrating decreased apoptosis in oocytes from older mice upon the removal of cumulus cells prior to IVM (Perez & Tilly 1997). Collectively these studies suggest that ageing of the oocyte’s somatic support cells may contribute to age-related defects in oocytes.

Finally, there are conflicting results that contradict any role for somatic nurse cells in oocyte function with age. The co-culture of oocytes from reproductively aged women with granulosa cells from younger women was not sufficient to improve aneuploidy or maturation rates of older oocytes (Esbert *et al.* 2021). Similarly, Gordon *et al.* (2023) demonstrated that the co-culture of oocytes with cumulus cells during rescue IVM did not significantly improve spindle integrity and chromosome alignment in MII oocytes or cleavage and blastulation after parthenogenic activation (Gordon *et al.* 2023). It is however important to note that both experimental systems lacked the formation of TZPs that could directly transport metabolites to the oocyte. Furthermore, the oocyte interaction with the young cumulus cells was limited to the period of meiosis rather than in the growing follicular environment where TZP interactions are more pronounced (Barrett & Albertini 2010, Abbassi *et al.* 2021). Taken together, it is not certain whether damage to the aged oocyte during the follicular stage is too advanced for co-culture during oocyte maturation to have an impact on age-related aneuploidy. This may not be surprising considering cohesin loss likely precedes this intervention. These interactions between granulosa or cumulus cells and the oocyte will be important for future work and offer an interesting paradigm to explore the concept of cell autonomous or non-autonomous ageing.

## Future perspectives

This review highlights the relationships between altered cellular metabolism and chromosome segregation defects in oocyte ageing. Declining mitochondrial function, ATP production and elevated ROS are likely to play a role in modulating cohesin levels, microtubule stability and the fidelity of the SAC. Understanding how oxidative stress induces cohesin depletion and a permissive SAC in mammalian oocytes needs further examination. There is a growing list of metabolic changes that may account for this, including declining metabolic support from cumulus cells, reductions in NAD^+^ metabolism, the polyamine spermidine and changes in the mevalonate pathway, with more that are likely to be revealed. It will be interesting to investigate whether there are changes in additional metabolic pathways, for example, GTP production from the TCA cycle, as other potential causes of aneuploidy during reproductive ageing. In addition, understanding how alterations in the metabolism of other macromolecules, including proteins, may also provide further insight into how chromosome segregation can be influenced in oocytes of advanced reproductive age. Understanding the molecular mechanisms that cause loss of oocyte integrity with age are important as age-related infertility has a major impact on women and society more generally and the medical practices attempting to address age-related infertility.

## Declaration of interest

RBG is an Associate Editor of *Reproduction* but has had no part in the reviewing of this manuscript. LEW is a co-founder and director of Jumpstart Fertility, which aims to develop NAD^+^ precursors in the area of assisted reproduction.

## Funding

RBG’s laboratory is funded by an Investigator Fellowship (APP211024) from the National Health and Medical Research Councilhttp://dx.doi.org/10.13039/501100000265 of Australia and by a gift from Open Philanthropy. ALM is funded by Wellcome through a Collaborative award (215625), an Investigator award (220780) and core funding for the Wellcome Centre for Cell Biology (203149). LEW is supported by an American Federation for Aging Researchhttp://dx.doi.org/10.13039/100000965 (AFAR)/Hevolution Investigator in Aging Biology, with lab funding through a Longevity Impetus Grant from Norn Group, and an NHMRC Development grant (APP2000211).

## Author contribution statement

BPM, ALM LEW and RBG all contributed to drafting and reviewing the manuscript.
